# A new antiproliferative noscapine analogue: chemical synthesis and biological evaluation

**DOI:** 10.18632/oncotarget.9642

**Published:** 2016-05-26

**Authors:** Peter E. Ghaly, Rabab M. Abou El-Magd, Cassandra D. M. Churchill, Jack A. Tuszynski, F. G. West

**Affiliations:** ^1^ Department of Chemistry, University of Alberta, Edmonton, AB T6G 2G2, Canada; ^2^ Department of Oncology, University of Alberta, Edmonton, AB T6G 1Z2, Canada; ^3^ Department of Physics, University of Alberta, Edmonton, AB T6G 2E1, Canada; ^4^ Genetic Engineering and Biotechnology Institute, City of Scientific Research and Technological Application, New Borg El-Arab City, Alexandria, 21934, Egypt

**Keywords:** noscapine, tubulin, microtubules, fluorescence quenching, docking

## Abstract

Noscapine, a naturally occurring opium alkaloid, is a widely used antitussive medication. Noscapine has low toxicity and recently it was also found to possess cytotoxic activity which led to the development of many noscapine analogues. In this paper we report on the synthesis and testing of a novel noscapine analogue. Cytotoxicity was assessed by MTT colorimetric assay using SKBR-3 and paclitaxel-resistant SKBR-3 breast cancer cell lines using different concentrations for both noscapine and the novel compound. Microtubule polymerization assay was used to determine the effect of the new compound on microtubules. To compare the binding affinity of noscapine and the novel compound to tubulin, we have done a fluorescence quenching assay. Finally, in silico methods using docking calculations were used to illustrate the binding mode of the new compound to α,β-tubulin. Our cytotoxicity results show that the new compound is more cytotoxic than noscapine on both SKBR-3 cell lines. This was confirmed by the stronger binding affinity of the new compound, compared to noscapine, to tubulin. Surprisingly, our new compound was found to have strong microtubule-destabilizing properties, while noscapine is shown to slightly stabilize microtubules. Our calculation indicated that the new compound has more binding affinity to the colchicine-binding site than to the noscapine site. This novel compound has a more potent cytotoxic effect on cancer cell lines than its parent, noscapine, and hence should be of interest as a potential anti-cancer drug.

## INTRODUCTION

**Figure 1:**
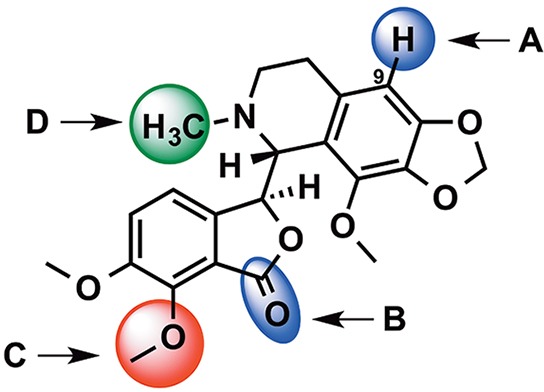
Structural modification of noscapine **A.** and **B.** represent sites of modification of the first generation noscapinoids. Second and third generation noscapinoids were generated by modifications at sites **C.** and **D.** respectively.

Noscapine, a phthalide isoquinoline alkaloid, is a natural product that was first isolated and characterized in 1817 by Pierre-Jean Robiquet [1] from the opium poppy, *Papaver somniferum*. Unlike other opium alkaloids, noscapine is non-addictive, non-narcotic and non-analgesic. It is widely used in many countries as an antitussive (cough suppressant) agent and has a low toxicity profile [2]. In 1998, the Joshi group found that noscapine possesses anticancer activity due to its action on tubulin [3]. As a tubulin-binding agent, noscapine has some pharmacological advantages. Noscapine was found to be effective in slowing tumour growth while having little toxicity in normal tissues [4], is effective in multidrug resistant cell lines [5, and has a favorable pharmacokinetic profile [6]. Noscapine is also known to trigger apoptosis in different cancer cell lines through the activation of different apoptotic pathways [7–10]. Over the last decade, many noscapine analogues have been synthesized and tested, showing anti-cancer activity superior to the parent noscapine. These analogues are synthesized by chemically modifying the parent noscapine molecule, while keeping the scaffold intact. The first generation noscapinoids were generated by chemically modifying the isoquinoline and benzofuranone rings of noscapine. This includes the 9′-halogenated (chloro-, bromo- and iodo-noscapine) [11], 9′-amino [12], 9′-nitro [13] and the 9′-azido analogues [14]. The first generation also includes cyclic ether halogenated analogues [15]. O-alkylated and O-acylated analogues represent the second-generation noscapinoids that were generated by modifying the benzofuranone ring of noscapine [16]. Third-generation noscapinoids were synthesized by modifying the substituents coupled to the nitrogen of the isoquinoline ring (Figure 1) [17].

Noscapine binds to tubulin stoichiometrically [18] to induce a conformational change in the protein [3], as found for other anti-mitotic agents that target tubulin [19, 20]. Noscapine is unique from other antimitotic agents since it has no significant effect on microtubule stabilization or destabilization [5], but instead alters the dynamic instability of microtubules by increasing the time spent in the pause phase [5]. Similar structural features between noscapine and colchicine, a known destabilizing agent [21], initially suggested these compounds may bind to the same site, although experiments found that noscapine does not compete with colchicine for binding to tubulin [3]. Interestingly, a small modification altering noscapine to 9-bromonoscapine results in a compound that disrupts colchicine binding [22], and slightly inhibits microtubule polymerization [23]. Therefore, understanding how noscapine and its analogues bind to and affect tubulin and microtubules has proven challenging without crystal structures or hydrogen-deuterium exchange mass spectrometry.

In 2011, using computational docking and molecular dynamics methods, noscapine was predicted to bind to a unique site on β-tubulin at the intradimer interface that is near the colchicine site, but does not interfere with colchicine binding [24]. This result was supported by competitive binding experiments showing a lack of competition between noscapine and colchicine [3]. Based on this newly identified binding site, a new library of noscapine analogues was proposed, which were computationally predicted to have higher affinity towards tubulin than noscapine [24]. These newly proposed analogues share a common scaffold within their structures. In our subsequent attempts to synthesize this common scaffold, we came across an interesting compound that showed promising anti-proliferative activity compared to noscapine.

In this study, we report the effect of this novel compound 8, on SKBR-3 breast cancer cells, its affinity towards tubulin, as well as its effects on microtubule polymerization. We have also studied the binding of this compound to various sites on tubulin using *in silico* methods.

## RESULTS

### Synthetic pathway for the new compound (8)

**Scheme 1:**
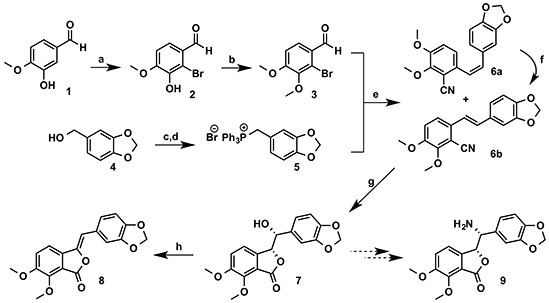
Preparation of compound 8. Reagents and conditions: **a.** Br_2_, Fe powder, NaOAc, AcOH, 1.5 h (83%); **b.** NaH, CH_3_I, DMF, rt, 15 h (76%); **c.** PBr_3_, DCM, rt, 2 h (96%); **d.** PPh_3_, toluene, rt, 3.5 h (97%); **e.** *n*-BuLi, THF, 0°C (30 min) - rt (14 h), then CuCN, DMF, reflux, 16 h (**6a**, 48% and **6b**, 32%); **f.** PdCl_2_(PPh_3_)_2_, (EtO)_3_SiH, THF, reflux, 15 h (85%); **g.** K_2_Fe(CN)_6_, K_2_CO_3_, (DHDQ)_2_PHAL, K_2_OSO_4_. 2 H_2_O, THF, *t*-BuOH, H_2_O (70%); **h.** TsCl, pyridine, DCM, rt, 3 h (65%).

**Figure 2:**
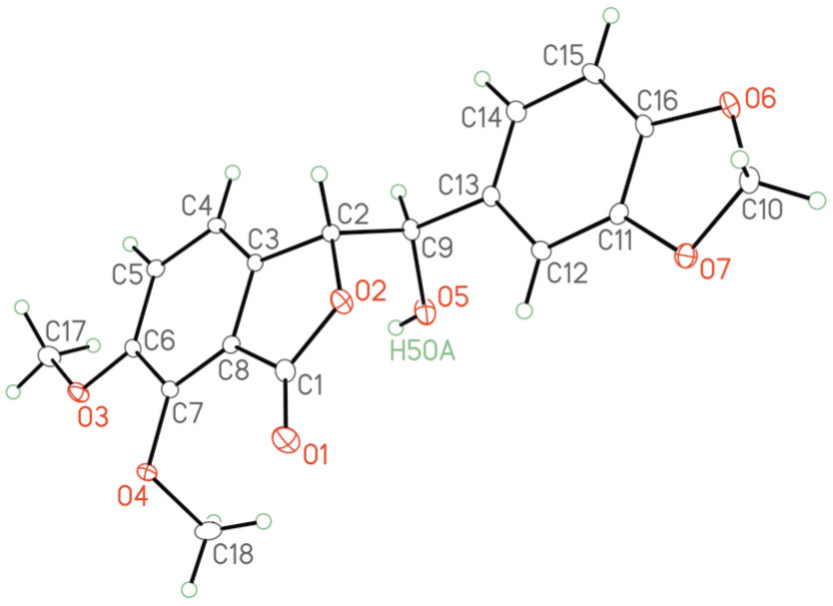
X-ray crystal structure for the alcohol 7.

Our synthesis (Scheme 1) began with the commercially available isovanillin 1. Regioselective bromination of 1 gave the 2-bromoisovanillin 2 [25] in 83% yield. This was followed by methylation of the phenolic hydroxyl group in 2 to give the 2-bromo-3,4-dimethoxybenzaldehyde 3 [26] in 76% yield. The phosphonium salt 5 was synthesized from the piperonyl alcohol 4 according to the literature procedure [27] in 93% overall yield. Compounds 3 and 5 were then coupled under Wittig reaction conditions to give inseparable *E/Z* olefin mixture, which was then treated with CuCN to afford a separable mixture of 6a/6b in 80% global yield with 60:40 ratio in favor of 6a [28]. The *Z*-isomer 6a was converted to the desired *E*-isomer using a palladium catalyzed isomerisation process [29]. Compound 6b was then converted exclusively to the enantiomerically pure (>99% ee) phthalide 7 via Sharpless asymmetric dihydroxylation using AD mix-β followed by *in-situ* cyclization with the cyano group [28]. We were also able to obtain an X-ray crystal structure^1^ for 7 (Figure 2). Conversion of 7 to the target molecule 9 via a sequence of tosylation, azide displacement and reduction failed, and only the undesired compound 8 was isolated in 65% yield. It is worth mentioning that treatment of 7 with triflic anhydride in pyridine or diphenyl phospheryl azide (DPPA) led to the formation of 8 in comparable yield.

### The effect of the new compound (8) on MT polymerization

**Figure 3:**
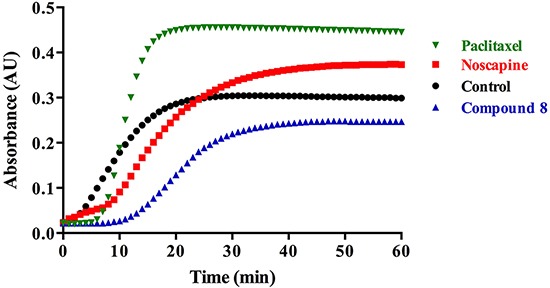
Microtubule assembly assay in the presence of noscapine, compound 8 or paclitaxel

To determine the effect of noscapine and compound 8 upon the assembly of tubulin subunits into microtubules, changes in the turbidity of tubulin solution were measured in the absence or presence of the tested compounds. The control (tubulin in the prepared buffer with DMSO) represents the normal polymerization of microtubules in the absence of any added compounds at 37°C (Figure 3). Paclitaxel, a known microtubule stabilizer, is used to represent MT polymerization. Noscapine is known to stabilize MT leading to their polymerization [3], however to a lesser extent compared to paclitaxel (Figure 3). We were expecting compound 8 to have a similar effect on MT polymerization as noscapine, however it was found to destabilize MT (Figure 3). These results suggest a different mechanism of action for compound 8 than noscapine.

### Binding affinity of the new compound (8)

**Figure 4:**
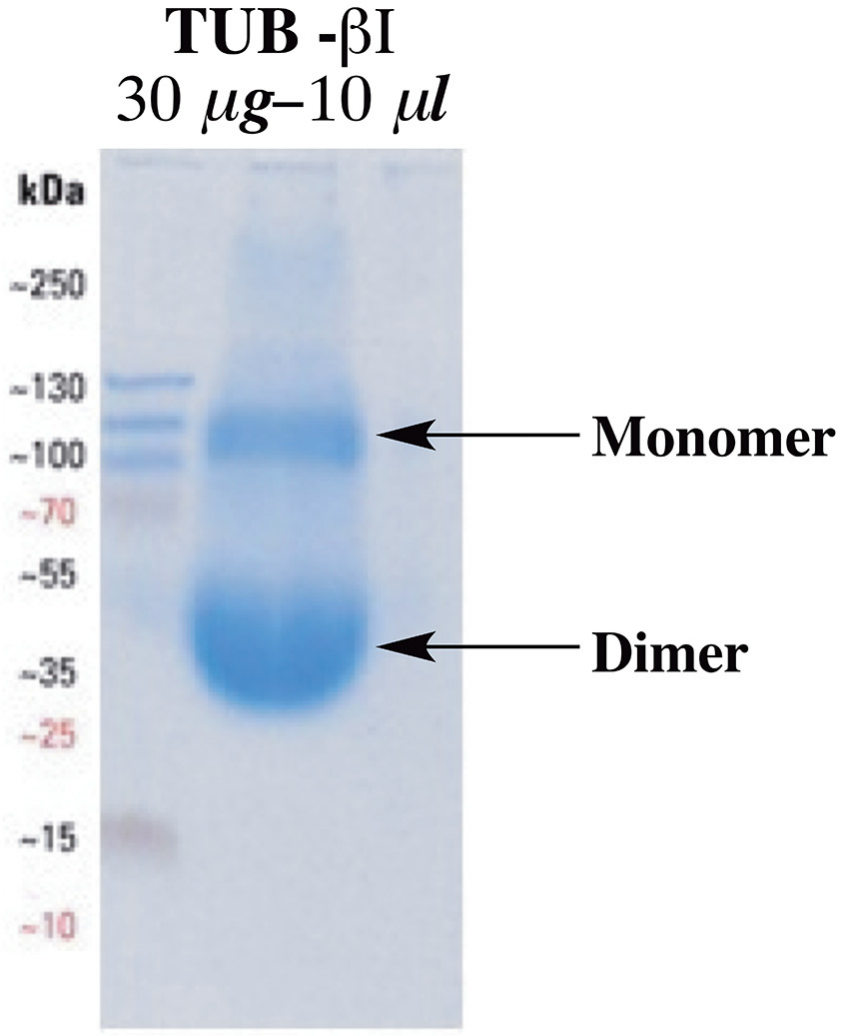
Native gel electrophoresis for the purified recombinant βI-tubulin at neutral pH using non-reducing loading buffer.

To test whether noscapine and compound 8 interact directly with tubulin, the fluorescence of α,β-tubulin heterodimers was examined in the presence and absence of noscapine, as well as compound 8. Interestingly, recombinant purified βI-tubulin was found to form homodimers, which gave the same characteristic bell-shaped tryptophan fluorescence with significant quenching in the presence of different concentrations of the tested compounds. The homodimer formation was confirmed by running native gel electrophoresis using a 10 μL solution containing 30 μg of the purified recombinant βI-tubulin (Figure 4).

**Figure 5:**
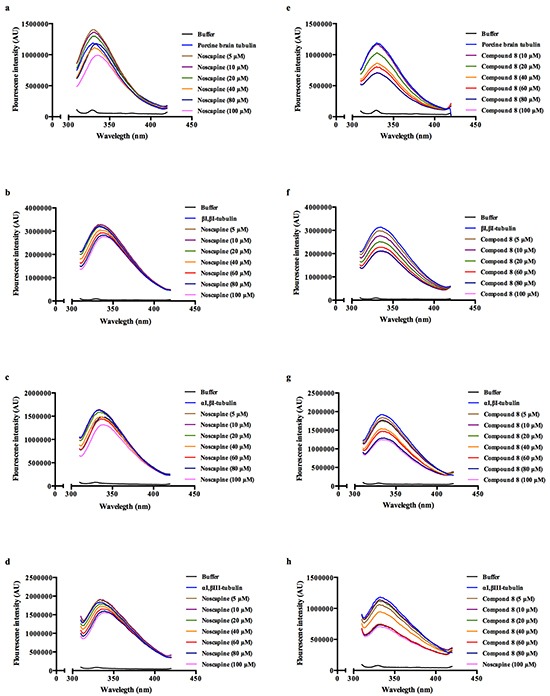
Fluoresence intensity quenching of noscapine **a–d.** and compound 8 **e–h.** using porcine brain tubulin (a,e), βI,βI-tubulin (b,f), αI,βI-tubulin (c,g) and αI,βIII-tubulin (d,h).

The effect of both noscapine and compound 8 were tested on αI,βI-tubulin heterodimers, αI,βIII-tubulin heterodimers, βI,βI-tubulin homodimers and porcine brain tubulin (unfractionated) to observe if there are any isotype-specific effects. Both compounds displayed notable quenching of tryptophan fluorescence in a concentration-dependent manner (Figure 5 and Table 1); however compound 8 showed a stronger quenching profile. These fluorescence quenching studies indicated that the ability of compound 8 to induce conformational changes upon binding varies depending on the tubulin isoform. The αI,βIII-tubulin isoform was found to be particularly affected, and it should be noted that βIII-tubulin is highly expressed in resistant tumor cells [30].

**Table 1:** Calculated binding affinity parameters; association (K_a_, 10^3^ M) and dissociation (K_d_, μM) constants for noscapine and compound 8 with porcine brain tubulin and purified recombinant tubulin dimers (βI,βI-tubulin, αI,βI-tubulin and αI,βIII-tubulin) determined using a fluorescence quenching assay

Compound Name	K_a_ (10^3^ M) and K_d_ (μM)							
	Porcine brain tubulin		βI,βI-tubulin		αI,βI-tubulin		αI,βIII-tubulin	
	K_a_	K_d_	K_a_	K_d_	K_a_	K_d_	K_a_	K_d_
Noscapine	3.77 ± 0.02	265.25	2.35± 0.04	425.53	3.41 ± 0.04	293.25	3.46 ± 0.01	289.02
Compound 8	5.75 ± 0.02	114.28	6.12 ±	163.39	5.78 ± 0.03	173.01	8.28 ± 0.10	121.06

### Antiproliferative effect of the new compound (8)

**Figure 6:**
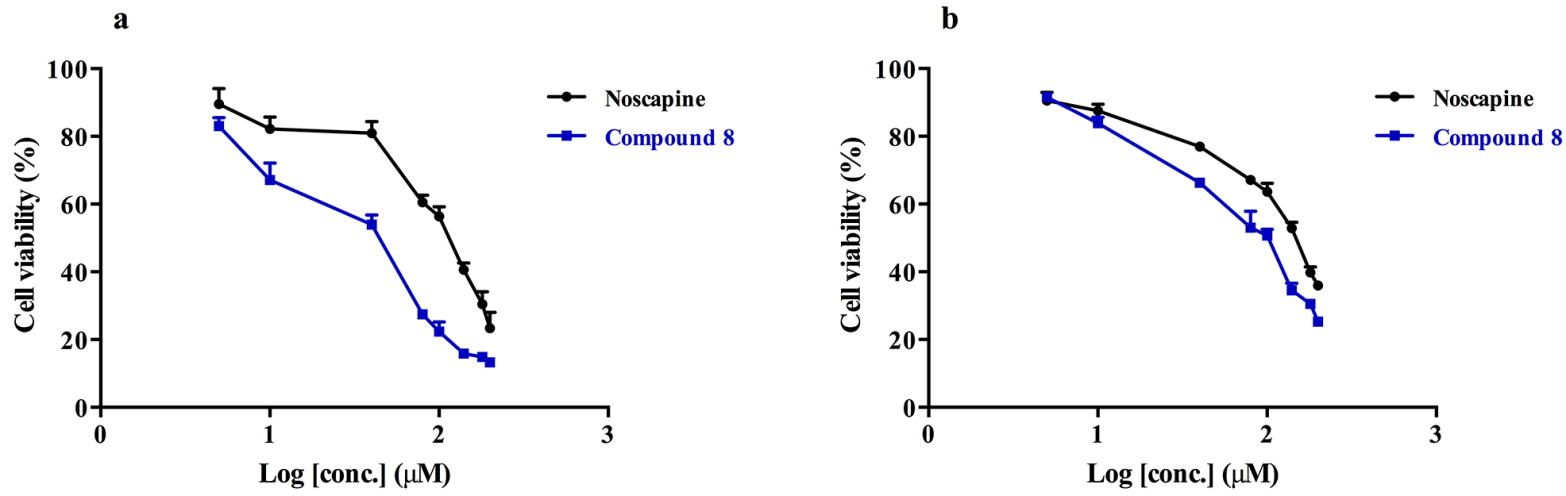
The effect of noscapine and compound 8 on the viability of breast cancer cell line **a.** SKBR-3; and **b.** paclitaxel resistant SKBR-3 using an MTT assay. Statistical analysis showed a statistically significant difference in the cytotoxicity of the compounds on both cell lines (p value < 0.05).

Arresting breast cancer cell growth and viability is still a challenge especially in view of drug resistance [31, 32], which calls for the development of appropriate new modalities of treatment. The effect of noscapine and compound 8 (Figure 6) on the viability of the human breast cancer cell lines SKBR-3, and paclitaxel-resistant SKBR-3 was investigated using the colorimetric MTT assay. This was motivated by the earlier studies discussed above that indicated noscapine may be suitable for drug development towards cancer chemotherapy with relatively low toxicity compared to other anti-mitotic agents. Our data revealed that compound 8 was more cytotoxic than noscapine on the SKBR-3 cell line with an IC_50_ of ~40 μM compared to ~100 μM for noscapine (Figure 6a). The same effect was also observed when using the paclitaxel-resistant SKBR-3, where compound 8 showed an IC_50_ of ~64 μM compared to ~100 μM for noscapine (Figure 6b).

### Determination of the binding site of the new compound (8) on tubulin

The above experimental results indicate that compound 8 is a microtubule-destabilizing agent (Figure 3), and therefore affects microtubules differently than noscapine. Although compound 8 is structurally similar to noscapine, it also shares some similarity with the microtubule-destabilizing agents colchicine and combretastatin A4, both of which are thought to bind to the colchicine domain located at the intra-dimer interface of αβ-tubulin [33, 34]. Furthermore, compound 8 has several features that match the pharmacophore for the colchicine site that was developed by Nguyen *et al.* based on the binding of colchicine, combretastatin A4 and other agents [35]. Therefore, we performed docking simulations to investigate the binding mode and the binding strength of these compounds to the colchicine binding site, and establish similarities in binding poses that may provide support for compound 8 binding to this site.

**Figure 7:**
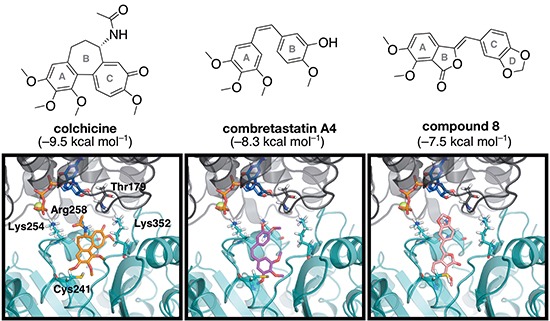
The energy-minimized docking poses of colchicine (orange), combretastatin A4 (magenta) and compound 8 (pink) in the colchicine binding domain located at the interdimer interface between α-tubulin (grey) and β-tubulin (teal). The nearby GTP and Mg^2+^ are shown in blue and yellow, respectively. Select residues are shown in stick mode, and labeled according to the numbering in the 1SA0 crystal structure. Docking scores (kcal mol^−1^) are indicated in brackets.

Docking scores indicate that colchicine binds with the greatest affinity, followed by combretastatin A4, while compound 8 has the lowest affinity for tubulin (Figure 7). The top-ranked docking poses for colchicine resemble the crystal structure pose, providing confidence in our docking protocols. Small variations exist in the orientation of the acetamide relative to the crystal structure, which has been previously shown to have high mobility in the binding site [36]. The top poses of the other ligands are also similar, which indicates a common binding motif can be established.

A comparison of the energy-minimized top docking pose for each of the three compounds indicates some variability (Figure 7). Both combretastatin A4 and compound 8 bind deeper into β-tubulin than colchicine, which supports previous work that found flexible ligands bind more deeply [36]. For each compound, the methoxy-containing A ring is directed into β-tubulin near Cys241, and overlap of these rings is observed for the compounds studied, as previously found for colchicine and combretastatin A4 [35, 37, 38]. The colchicine A ring has been identified as an essential feature of the pharmacophore [39]. However, no direct hydrogen bonds form between the protein and these methoxy groups. It is possible stability is gained from an S-H···O or S−H···π interaction between Cys241 and the A ring of the ligands. Interactions occur between the ligands and residues Lys254 and Lys352; ligand lone pairs are directed towards the lysine side chain amino group in the binding poses for all three ligands (Figure 7). However, colchicine is positioned closest to these lysine residues, compared to the other ligands. Arg258 is also in a position to interact with the ligands. These features indicate that compound 8 binds to the colchicine site in a fashion similar to that of other known colchicine-domain ligands.

## DISCUSSION

This paper reports the results of synthesis, *in vitro* testing and *in silico* modeling of a novel noscapine analogue **8**. Noscapine has been repurposed from its original application as an anti-tussive agent to a cancer chemotherapy, particularly as a second line of treatment [40]. Unfortunately, while showing low toxicity, it also failed to demonstrate sufficient efficacy in clinical trials [41], although it shows some promise as a prophylactic agent [42]. In this paper, we focused on an analogue of noscapine that was synthesized in the hope of improving its cytotoxicity profile compared to the parent compound.

In MTT assays involving both SKBR-3 and the paclitaxel-resistant SKBR-3 breast cancer cell line, both noscapine and compound 8 show cytotoxic activity in the sub-mM range, with compound 8 being demonstrably more potent. Noscapine had IC_50_ of ~100 for both cell lines, however compound 8 showed lower IC_50_ values of ~40 μM and 64 μM for the normal and the resistant SKBR-3 cell lines respectively. These cytotoxicity results were confirmed by the fluorescence quenching assays, which showed that compound 8 has lower K_d_ values, thus higher binding affinity, than its parent noscapine towards tubulin. The fluorescence quenching assays were done on porcine brain tubulin as well as purified recombinant tubulin dimers (βI,βI-tubulin, αI,βI-tubulin and αI,βIII-tubulin). All tubulin isoforms showed similar results confirming the stronger binding of compound 8 towards tubulin. To determine the effect of compound 8 on microtubules, whether it stabilizes or destabilizes their polymerization, we did a MT polymerization assay using both compound 8 as well as noscapine. Noscapine is a known MT stabilizer that enhances the polymerization of MT. Interestingly, in contrast to noscapine, we found that compound 8 has strong microtubule-destabilizing properties. This surprising result suggest a different mechanism of action for compound 8, which might be due to a different binding site on the α,β-tubulin protein.

To have a deep insight on the binding site and mode of this compound to α,β-tubulin, we performed docking calculations for both compound 8 and noscapine towards both the colchicine as well as the noscapine binding sites. Theses calculations have illustrated the binding mode of compound 8 to α,β-tubulin at the colchicine binding site, which we have shown is similar to that of other colchicine domain binders. This finding is consistent with structural features of compound 8 that have strong similarity with colchicine. It appears, therefore, that starting from the noscapine scaffold one can design compounds that gradually lose affinity for the noscapine-binding site and acquire propensity to bind to the colchicine binding site. Concomitant with this, there is a change in the mode of action of the compound, from stabilizing microtubules to destabilizing microtubules. It's worth mentioning that although compound 8 possesses low potency, it can be used in combination with other chemotherapeutic agents (paclitaxel) due to its low toxicity to get a synergistic effect and overcome cancer resistance. Similar effects were observed for paclitaxel when used in combination with the reduced 5-bromonoscapine analogue [43, 44]. Therefore further exploration of this new scaffold is required for the development of more potent tubulin binders.

## MATERIALS AND METHODS

### Materials

Noscapine and guanosine 5′-triphosphate (GTP) sodium salt hydrate were purchased from Sigma Aldrich, Canada Co. The noscapine stock solution was prepared at 2 mM in dimethyl sulfoxide (DMSO) and kept at −20°C. Porcine brain tubulin (Cat.# T240-DX) was purchased from Cytoskeleton Inc. The genes for human αI-, βI- and βIII-tubulin were purchased from DNA2.0 (Menlo Park, CA, USA). All reagents were purchased from Sigma-Aldrich Canada Ltd. (Oakville, Ontario, Canada) and Fisher Scientific Company (Ottawa, Ontario, Canada). Nickel-NTA resin was purchased from Qiagen Inc. (Toronto, Ontario, Canada).

### Methods

#### General procedure for chemical synthesis

Reactions were carried out in flame-dried glassware under a positive argon atmosphere unless otherwise stated. Transfer of anhydrous solvents and reagents was accomplished with oven-dried syringes or cannulae. Solvents were distilled before use: dichloromethane (CH_2_Cl_2_) and dimethylformamide (DMF) from calcium hydride, tetrahydrofuran (THF), and toluene from sodium/benzophenone ketyl and pyridine from KOH. Thin layer chromatography was performed on glass plates precoated with 0.25 mm silica. Flash chromatography columns were packed with 230–400 mesh silica gel. Optical rotations were measured using a Perkin Elmer 241 Polarimeter at 22 ± 2°C. Proton nuclear magnetic resonance spectra (^1^H NMR) were recorded at 500 MHz and coupling constants (J) are reported in hertz (Hz). Standard notation was used to describe the multiplicity of signals observed in ^1^H NMR spectra: broad (br), multiplet (m), singlet (s), doublet (d), triplet (t), etc. Carbon nuclear magnetic resonance spectra (^13^C NMR) were recorded at 125 MHz and are reported (ppm) relative to the centerline of the triplet from chloroform-d (77.0 ppm), or the centerline of the heptuplet from methanol-d_4_ (49.0 ppm). Infrared (IR) were measured using a Thermo Nicolet 8700 main bench with an attached Continuum FTIR microscope. Mass spectra were determined on a high-resolution electrospray positive ion mode spectrometer. Melting points were measured using the Thomas Hoover Capillary Melting Point Apparatus.

#### Procedure for the synthesis of 2-bromo-3,4-dimethoxybenzaldehyde (3)

NaH (1.32 g, 32.9 mmol) was added to a stirred solution of 2-bromo-3-hydroxy-4-methoxybenzaldehyde **2** [25] (6.30 g, 27.4 mmol) in anhydrous DMF (80 mL) at 0°C for 15 min. CH_3_I (2.05 mL, 32.9 mmol) was then added as a single portion to the reaction mixture and left to stir at room temperature for 15 h. The solvent was evaporated under reduced pressure to give the crude product, which was then dissolved in CH_2_Cl_2_, washed with water, brine, and dried over Na_2_SO_4_. The organic layer was filtered, concentrated under reduced pressure, and then purified by column chromatography on silica gel using 20% EtOAc/hexane as the eluent to afford **3** (5.07 g, 20.8 mmol, 76% yield) as a white solid that matched previously reported characterization data [26]: ^1^H NMR (500 MHz, CDCl_3_) δ 10.26 (d, *J =* 0.5 Hz, 1H), 7.75 (d, *J =* 11.0 Hz, 1H), 6.96 (d, *J =* 11.0, 0.5 Hz, 1H), 3.96 (s, 3H), 3.89 (s, 3H).

#### Procedure for the synthesis of *(E/Z)*-6-(2-(benzo[*d*][1,3]dioxol-5-yl)vinyl)-2,3-dimethoxybenzonitrile (6a/6b)

*n*-Butyllithium in hexane (2.1 M, 7.01 mL, 14.7 mmol) was added dropwise to a stirred solution of **5** [27] (6.99 g, 14.7 mmol) in anhydrous THF (30 mL) at 0°C. The solution was stirred for 30 min at 0°C, then 2-bromo-3,4-dimethoxybenzaldehyde **3** (3.41 g, 14.0 mmol) in THF (15 mL) was added dropwise via syringe at the same temperature. The reaction mixture was allowed to stir for 14 h at room temperature (monitored by TLC). The reaction mixture was then cooled to 0°C, and saturated solution of NH_4_Cl (25 mL) was added. The aqueous layer was separated and extracted with CH_2_Cl_2_ (3 × 25 mL). The organic layers were combined, washed with water, brine, dried over anhydrous Na_2_SO_4_, filtered, and concentrated under reduced pressure to give the crude product as a mixture of E/Z. The crude product was then dissolved in DMF (40 mL) at room temperature. CuCN (1.88 g, 21.0 mmol) was then added to the reaction mixture, which was then refluxed for 16 h. The reaction mixture was then cooled down to room temperature before adding H_2_O (20 mL). Next, the aqueous layer was separated and extracted with CH_2_Cl_2_ (3 × 25 mL). The organic layers were combined, washed with water, brine, dried over anhydrous Na_2_SO_4_, and concentrated under reduced pressure then purified by column chromatography on silica gel using 30% EtOAc/hexane as the eluent to afford both *E-* and *Z-* isomers in 48% (2.07 g, 6.72 mmol) and 32% (1.38 g, 4.48 mmol) isolated yields respectively.

#### *(Z)*-6-(2-(benzo[*d*][1,3]dioxol-5-yl)vinyl)-2,3-dimethoxybenzonitrile (6a)

Pale yellow oil; R*_f_* = 0.30 (70:30 Hexane: Ethyl acetate); IR (cast film) ν_max_ = 3011, 2944, 2900, 2840, 2227, 1595, 1565, 1492, 1446, 1417, 1353, 1266, 1239, 1214, 1194, 1180, 1091, 1073, 1039 cm^−1^; ^1^H NMR (500 MHz, CDCl_3_) δ 7.02 (dd, *J* = 8.5, 0.5 Hz, 1H), 6.92 (d, *J* = 8.5 Hz, 1H), 6.67-6.66 (m, 2H), 6.60 (d, *J* = 12.0 Hz, 1H), 6.60-6.59 (m, 1H), 6.5 (d, *J* = 12.0 Hz, 1H), 5.87 (s, 2H), 3.98 (s, 3H), 3.83 (s, 3H); ^13^C NMR (125 MHz, CDCl_3_) δ 151.7, 151.2, 147.5, 147.1, 133.4, 132.7, 130.2, 125.0, 124.3, 123.3, 116.6, 115.1, 108.6, 108.3, 107.4, 101.1, 61.6, 56.1; HRMS (ESI) calcd for C_18_H_15_NNaO_4_ [M + Na]^+^ 332.0893; found 332.0892.

#### *(E)*-6-(2-(benzo[*d*][1,3]dioxol-5-yl)vinyl)-2,3-dimethoxybenzonitrile (6b)

White solid; mp 129-131°C; R*_f_* = 0.21 (70:30 Hexane: Ethyl acetate); IR (cast film) ν_max_ = 3005, 2943, 2903, 2841, 2225, 1632, 1604, 1593, 1565, 1494, 1449, 1416, 1361, 1295, 1278, 1253, 1233, 1198, 1124, 1098, 1074 cm^−1^; ^1^H NMR (500 MHz, CDCl_3_) δ 7.41 (d, *J* = 9.0 Hz, 1H), 7.14 (d, *J* = 16.0 Hz, 1H), 7.11 (d, *J* = 9.0 Hz, 1H), 7.08 (d, *J* = 1.5 Hz, 1H), 7.04 (d, *J* = 16.0 Hz, 1H), 6.96 (dd, *J* = 8.0, 1.5 Hz, 1H), 6.80 (d, *J* = 8.0 Hz, 1H), 5.99 (s, 2H), 4.03 (s, 3H), 3.91 (s, 3H); ^13^C NMR (125 MHz, CDCl_3_) δ 151.7, 151.3, 148.3, 148.0, 133.6, 131.2, 131.0, 122.1, 122.0, 120.5, 117.2, 115.2, 108.5, 106.5, 105.8, 101.3, 61.7, 56.3; HRMS (ESI) calcd for C_18_H_15_NNaO_4_ [M + Na]^+^ 332.0893; found 332.0895.

#### Procedure for the synthesis of (R)-3-((R)-benzo[*d*][1,3]dioxol-5-yl(hydroxy)methyl)-6,7-dimethoxyisobenzofuran-1(3H)-one (7)

K_3_Fe(CN)_6_ (3.68 g, 11.2 mmol) and K_2_CO_3_ (1.55 g, 11.2 mmol) were added to a solution of *t*-BuOH (10 mL), THF (10 mL) and H_2_O (20 mL) and stirred for 10 min at room temperature. (DHQD)_2_PHAL [28] (26.5 mg, 1.0 mol%) and K_2_OsO_4_·2H_2_O (12.5 mg, 1.0 mol%) were then added and stirring of the mixture was continued for 30 min at room temperature. To the stirring reaction mixture was then added compound 6b (1.1 g, 3.4 mmol). After stirring for 18 h at room temperature, sodium bisulphite (3.0 g, 28.8 mmol) and H_2_O (10 mL) were added and the reaction mixture was stirred for further 2 h. The aqueous layer was then separated and extracted with CH_2_Cl_2_ (3 × 25 mL). The organic layers were combined, washed with water, brine, dried over anhydrous Na_2_SO_4_, filtered and concentrated under reduced pressure then purified by column chromatography on silica gel using 40% EtOAc/hexane as the eluent to afford **7** (0.83 g, 2.4 mmol, 70% yield) as white solid; mp 153–155°C; >99% ee by chiral HPLC analysis (Chiracel AD-H, n-hexane–iPrOH, 80:20, 1 mL min^−1^) retention time 42.47 (>99%); R*_f_* = 0.67 (20:80 Hexane: Ethyl acetate); [α]_D_^20^+14.66 (*c* 0.15, DCM); IR (cast film) ν_max_ = 3479, 3068, 2934, 2852, 1759, 1598, 1501, 1444, 1425, 1350, 1272, 1252, 1194, 1165, 1117, 1099, 1037 cm^−1^; ^1^H NMR (500 MHz, CD_3_OD) δ 7.30 (d, *J* = 8.5 Hz, 1H), 6.87 (dd, *J* = 8.5, 1.0 Hz, 1H), 6.79 (d, *J* = 1.5 Hz, 1H), 6.75 (dd, *J* = 8.0, 1.5 Hz, 1H), 6.72 (d, *J* = 8.0 Hz, 1H), 5.91 (d, *J* = 1.0 Hz, 1H), 5.90 (d, *J* = 1.0 Hz, 1H), 5.53 (dd, *J* = 5.5, 1.0 Hz, 1H), 4.85 (d, *J* = 5.5 Hz, 1H), 3.90 (s, 3H), 3.86 (s, 3H), (OH proton could not be observed in CD_3_OD);^13^C NMR (125 MHz, CD_3_OD) δ 170.1, 154.1, 149.0, 148.9, 148.9, 141.2, 134.5, 122.2, 120.8, 120.1, 119.8, 108.8, 108.6, 102.4, 84.2, 75.7, 62.4, 57.3; HRMS (ESI) calcd for C_18_H_16_NaO_7_[M + Na]^+^ 367.0788; found 367.0785.

#### Procedure for the synthesis of (Z)-3-(benzo[*d*][1,3]dioxol-5-ylmethylene)-6,7-dimethoxyisobenzofuran-1(3H)-one (8)

*p*-Toluenesulfonyl chloride (0.13 g, 0.67 mmol) was added portionwise to a solution of **7** (0.21 g, 0.61 mmol) and pyridine (74.4 μL, 0.92 mmol) in DCM (10 mL) at room temperature. After stirring for 2 h at room temperature, water (5 mL) was added and the solution was extracted with CH_2_Cl_2_ (3 × 10 mL). The organic layers were combined, washed with water, brine, dried over anhydrous Na_2_SO_4_, filtered and concentrated under reduced pressure then purified by column chromatography on silica gel using 50% EtOAc/hexane as the eluent to afford **8** (0.13 g, 0.40 mmol, 65% yield) as yellow solid; mp 159-161°C; R*_f_* = 0.37 (60:40 Hexane: Ethyl acetate IR (cast film) ν_max_ = 3064, 3008, 2954, 2927, 2856, 1771, 1758, 1732, 1664, 1616, 1596, 1502, 1447, 1365, 1350, 1279,1258, 1198, 1167, 1139, 1127, 1109, 1076, 1040, 1026 cm^−1^;^1^H NMR (500 MHz, CDCl_3_) δ 7.51 (d, *J* = 2.0 Hz, 1H), 7.38 (d, *J* = 8.5 Hz, 1H), 7.28 (d, *J* = 8.5 Hz, 1H), 7.19 (dd, *J* = 8.5, 2.0 Hz, 1H), 6.85 (d, *J* = 8.5 Hz, 1H), 6.21 (s, 1H), 6.02 (s, 2H), 4.18 (s, 3H), 3.97 (s, 3H);^13^C NMR (125 MHz, CDCl_3_) δ 164.6, 152.9, 148.2, 148.1, 147.5, 142.8, 134.2, 127.8, 124.5, 120.0, 115.4, 114.4, 109.6, 108.5, 104.9, 101.3, 62.4, 57.0; HRMS (ESI) calcd for C_18_H_14_NaO_6_ [M + Na]^+^ 349.0683; found 349.0681.

#### Microtubule assembly assay

The turbidity was recorded on 96-half area well plates by microplate reader at 340 nm as an indicator for microtubules formation. The wells containing 80 mM piperazine-N,N’-bis[2-ethanesulfonic acid] sequisodium salt (PIPES buffer, pH 6.9); 2.0 mM MgCl_2_; 0.5 mM ethylene glycol-bis(β-amino-ethyl ether) N,N,N’,N’-tetra-acetic acid (EGTA), 10 μM of noscapine or compound 8 in DMSO were kept at room temperature. Tubulin at a concentration of 3mg/mL in tubulin buffer (80 mM PIPES pH 6.9, 2 mM MgCl_2_, 0.5 mM EGTA, 1 mM GTP, 10.2% glycerol) was kept at 4°C before being added to the wells and shifting to 37°C. The absorbance was measured using the kinetic absorbance mode. DMSO solutions of paclitaxel and colchicine (10 μM) were used as controls.

#### Preparation of human αI-, βI- and βIII-tubulin

The protein sequence of human αI-tubulin is given by UniProtKB accession number Q71U36 (gene name TUBA1A), the protein sequence of human βI-tubulin is given by UniProtKB accession number P07437 (gene name TUBB) and that of human βIII-tubulin is given by UniProtKB accession number Q13509 (gene name TUBB3). The cloning work for αI- and βI-tubulin was performed and reported previously [45]. For the βIII human tubulin protein, the sequence was converted into DNA sequences with codons optimized for production in *Escherichia coli*, and for purification purposes, a His-tag was added at the N-terminus. The βIII-tubulin gene was inserted into a pET15b vector between the XhoI and NdeI restriction sites. The correct sequence, insertion, and orientation of the tubulin constructs were verified by DNA sequencing. Recombinant proteins were expressed in *E. coli* BL21 (DE3) host cells in LB medium supplemented with 100 μg/mL ampicillin. The cultures were grown at 37°C until an OD600 = 0.8 was reached, and the cells were induced with 1.0 mM isopropyl b-D-1-thiogalactopyranoside (IPTG) for 18 h at 25°C. After induction, the cells were harvested by centrifugation (6000 × g for 20 min at 4°C in SLC-6000 evolution sorvall rotor). The three variants of the tubulin protein were isolated from the inclusion bodies.

The αI-, βI- and βIII-tubulin constructs were purified in the same manner *via* fast refolding by dilution with metal affinity chromatography (IMAC) using a Ni-NTA column. The cell pellets from 1 L of the LB medium with expressed tubulin protein were resuspended in 25 mL of buffer A (buffer A: 50 mM Tris, 50 mM MgSO_4_, 50 mM NaCl, pH 8.8) and lysed by sonication (using Fisher Scientific Ultrasonic Dismembrator Model 500 with microtip probe for 4 times (30 seconds each) pulses at 45% power) on ice followed by centrifugation at 12000 × g for 20 min (4°C) in JA 25–50 fixed angle rotor, Beckman Coulter centrifuge. The supernatant was removed, and the inclusion bodies were cleaned by a series of washing steps with buffer A containing 0.1% Triton X-100, 25% glycerol, 500 mM NaCl, and 2 M urea as a separate additive for every next wash. Inclusion bodies were centrifuged at 12,000 × g for 20 min (4°C) in JA 25–50 rotor after every wash, and the supernatant was removed. The clean protein pellet was solubilized in buffer B (buffer B: 50 mM Tri50 mM NaCl, 1 mM CaCl_2_, 8 M urea, 10 mM beta-mercapto- ethanol, pH 8.8) and left for slowly rotated incubation at room temperature overnight. The next day, the sample was centrifuged at 33,000 × g for 1 h (25°C) in a JA 25–50 rotor. The tubulin proteins were refolded via rapid dilution (1:10 volume/volume) into buffer C (buffer C: 50 mM Tris, 50 mM NaCl, 10 mM MgSO_4_, 1 mM CaCl_2_, pH 7.4) and loaded onto a Ni-NTA column (25 mL bed volume) pre-equilibrated with buffer C. The loaded sample was incubated on a column for 1 h (4°C) with rotation. The column was then washed with buffer D (buffer D: 50 mM Tris, 50 mM NaCl, 10 mM MgSO_4_, 1 mM CaCl_2_, 10 mM imidazole, pH 7.3), and tubulins were eluted with a linear gradient of 500 mM imidazole in buffer D. Fractions containing the protein were identified by spot testing and SDS–PAGE gel, then mixed followed by overnight dialysis (4°C with two buffer changes) against 10 volumes of buffer E (buffer E: 25 mM Tris, 25 mM NaCl, 10 mM MgSO_4_, 1 mM MgCl_2_, 1 mM CaCl_2_, pH 7.3). The protein concentration (μM) was determined using the corresponding extinction coefficient at 280 nm (αI-tubulin: 51060 M^−1^cm^−1^, βI-tubulin: 46340 M^−1^ cm^−1^ and βIII-tubulin: 47832 M^−1^ cm^−1^) calculated by the ProtParam software based on recombinant αI-, βI- and βIII-tubulin amino acid sequences. The three proteins were then concentrated using an Amicon Ultra-15 centrifugal device.

#### Binding experiments and tryptophan fluorescence quenching assays

In a 96-well microplate, equimolar mixtures of recombinant human tubulin monomers, as well as buffer (10 mM sodium phosphate, 10 mM MgCl_2_, 1 mM GTP, 0.5% DMSO, 250 mM sucrose, pH 7.0) were combined to reach a final tubulin dimer concentration of 2 μM for βI,βI-tubulin, αI,βI-tubulin and αI,βIII-tubulin. GTP was added to the samples to a final concentration of 1 mM. The microplate was incubated on ice for 10 min to allow for the formation of the tubulin dimer. The calculated amounts of stock solution of the compounds in DMSO were added to the protein samples to obtain final ligand concentrations of 5, 10, 20, 40, 60, 80 and 100 μM. The control was ligand-free, and the total sample volume was 100 μL. A glass bead was inserted into each well, and the microplate was covered with protective film, sealed with a lid, and incubated for 30 min at 25°C. After that time, the microplate was transferred to a rotating plate form and vigorously rotated for 1 h at room temperature. From each well, 80 μL of samples and control were transferred to a 1 cm fluorescence cell. Fluorescence spectra were collected on a PTI MODEL-MP1 spectrofluorometer using a 10 mm path length cell at 295 nm (excitation wavelength) and a scan range of 310–400 nm. Data analysis was performed using ORIGIN 6.1 software (Origin-Lab, Northampton, MA, USA).

#### Determination of binding affinity parameters

The apparent binding constant of noscapine and compound 8 to different tubulin isoforms was calculated using data from the fluorescence experiments via the Stern–Volmer equation [46]:

(F0−F)/F=Ka[L]a (1)

where *F_0_* and *F* are the fluorescence intensities in the absence and in the presence of quencher respectively, *K_a_* is the formation constant of the donor–acceptor (quencher–fluorogen) complex, and *[L]_a_* is the concentration of the tested compound added. Excitation and emission slits were set at 4 nm. All spectra were collected with samples having final optical densities (1 cm) < 0.3 at maximum absorbance of added ligand and were corrected for the inner filter effect according to equation 2 [47]:

$${\text{F}}_{\text{corr}}={\text{F}}_{\text{obs}}\times {\left[10\right]}^{\left({\text{A}}_{\text{ex}}+{\text{A}}_{\text{em}}\right)/2}$$ (1)

where *F_corr_* is the corrected fluorescence, *F_obs_* is the measured fluorescence, *A_ex_* is the absorption value at the excitation wavelength (295 nm), and *A_em_* is the absorption value at the emission wavelength (336 nm). From the slope of the linear plot of *((F_0_*– F*)/F)* versus *[L]_a_*, binding constants were estimated. The results are expressed as mean values SD (n=4).

#### Cell culture

The human breast cancer cell lines SKBR-3 and paclitaxel-resistant SKBR-3 were kindly provided by Marc St. George (University of Alberta, Canada) [48]. Both cell lines were grown in RPMI 1640 medium (GIBCO) with 10% fetal calf serum and 1 mM L-glutamine 1% penicillin/streptomycin mixture under a humidified atmosphere containing 5% CO_2_. Addition of 16.65 nM paclitaxel to the paclitaxel-resistant cell line is mandatory to keep the acquired resistance at the same efficiency level.

#### MTT assay

The human breast cancer cell lines SKBR-3 and paclitaxel-resistant SKBR-3 (1 × 10^4^ cells per well) were seeded into 96-well plates. After incubation for 24 h (when cells reached 70–80% confluency), the medium was aspirated and the cells were treated with several concentrations of noscapine, as well as compound 8. After 21 h incubation, 50 μL of MTT (1 mg/mL) solution was added and the plates were incubated for an additional 3 h. After centrifugation, supernatant was removed from each well and 150 mL of dimethyl sulfoxide (DMSO) was added to dissolve the insoluble formazan crystals. The absorbance was measured at 570 nm and 690 nm subtracted as a background, using microplate reader. The data was plotted using GraphPad Prism 5.0 software. IC_50_ and statistical analysis (t-test) were calculated using the same software.

#### Computational details of the docking calculations

Colchicine, combretastatin A4 and compound 8 were docked to the colchicine binding site. Receptor coordinates were obtained from the 1SA0 crystal structure [30] in the Protein Data Bank (PDB). To prepare the αβ-tubulin heterodimer for docking, hydrogen atoms were added by the tleap module of AmberTools [49] and the Protonate 3D tool in the Molecular Operating Environment (MOE) software program [50] Nucleotide cofactors and magnesium ions were retained. Subsequently, this complex was energy minimized using the Amber12:EHT force field in MOE.

Using the MOE program, compounds were docked to the receptor at the colchicine site identified in the 1SA0 structure. An induced fit protocol was used for docking calculations. The receptor was defined as the protein, nucleotide cofactors and Mg^2+^ ions. Receptor atoms belonging to residues within 4.5 Å from the crystalized colchicine coordinates were allowed to move during docking. Docking poses were first scored with the London dG method and the top 30 unique hits were rescored with the GBVI/WSA dG methods, where the top 10 unique hits were retained. Duplicate poses were discarded. Following the docking calculations, the ligand-receptor complex for the top pose of each compound was energy minimized using the Amber12:EHT force field in MOE to maximize ligand-receptor interactions.

## SUPPLEMENTARY MATERIALS



## Footnotes

1 A Crystallographic Information File (CIF) for the structure of compound 7 is available as supporting information. The crystallographic data are also available free of charge from the Cambridge Crystallographic Data Center (http://www.ccdc.cam.ac.uk) using the registry number CCDC 1433128.
